# Paraneoplastic pemphigus: Diagnostic mimics, confounders and management challenges in a series of five long‐term survivors from a single centre

**DOI:** 10.1002/ski2.200

**Published:** 2022-12-19

**Authors:** Jonathan S. Emerson, Mark Schifter, Pei Dai, Jocelyn Jiang, Mark S. Taylor, Michelle Kang, Kenelm Kwong, Hadleigh Clark, Suzanne Cullican, David Campbell, Ming‐Wei Lin

**Affiliations:** ^1^ Department of Clinical Immunology and Immunopathology Westmead Hospital Sydney New South Wales Australia; ^2^ Sydney Medical School University of Sydney Camperdown New South Wales Australia; ^3^ Department of Oral Medicine, Oral Pathology and Special Needs Dentistry Westmead Centre for Oral Health Westmead Hospital Sydney New South Wales Australia; ^4^ Sydney Dental School Faculty of Medicine and Health The University of Sydney Sydney New South Wales Australia

## Abstract

We present a series of five cases who presented to our institution with treatment‐refractory mucosal ulceration, all of whom were subsequently diagnosed with paraneoplastic pemphigus (PNP). This case series highlights the diagnostic and treatment considerations for PNP – in particular, the steroid‐dependent, recalcitrant, polymorphic manifestations; the combination of histopathological and clinical findings that may overlap with clinically similar diseases, for example, pemphigus vulgaris and lichen planus; the importance of immunopathological findings for its diagnosis, and the need for surveillance and management of life‐threatening bronchiolitis obliterans.

## Graphical abstract

Paraneoplastic pemphigus (PNP) is associated with a poor prognosis. This may be due to its invariable association with malignancy, the need for immunosuppression, as well as bronchiolitis obliterans, the latter of which has a high mortality rate. Good clinicopathologic correlation is key to its timely diagnosis. Here, we highlight the clinical features and diagnostic findings that portend PNP, and detail our institute’s experience with its management, which has resulted in an impressive long‐term survival even when compared to recent estimates of survival in the literature.

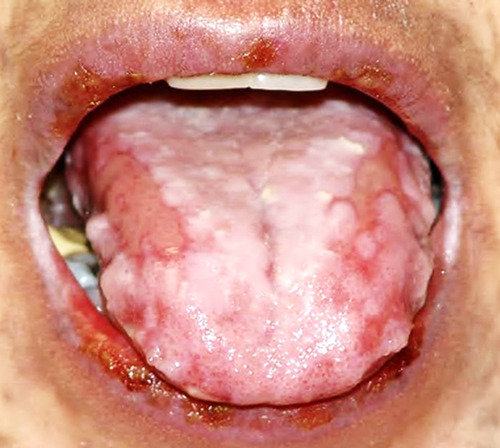

## 1 INTRODUCTION

Paraneoplastic pemphigus (PNP) is a rare autoantibody‐driven mucocutaneous disease that not only poses a diagnostic challenge, but is also characterised by a poor prognosis and high mortality rate.^1^ It presents similarly to other autoimmune bullous diseases (e.g., pemphigus vulgaris (PV) or bullous pemphigoid) and inflammatory dermatoses (e.g., lichen planus (LP)), which may delay diagnosis and appropriate treatment. PNP may, however, be distinguished by recalcitrance to conventional immunosuppression, an invariable association with malignancy (which may be subclinical), and its potential to cause life‐threatening pulmonary complications.

We outline our experience of five cases of PNP managed at the Oral Immunology Clinic at Westmead Hospital, including two cases previously reported in 2015,^2^ which highlight the clinical features and investigations that may lead to its early recognition and minimisation of morbidity (Table 1).

**TABLE 1:** Patients and clinical characteristics (*n* = 5)

Patient no.	Age/Sex/Ethnicity	Duration before PNP diagnosis (previous diagnosis)	Treatment prior to PNP diagnosis	Clinical features at presentation	Diagnosis of PNP			Treatment			Outcome
					Serology	Histopathology	DIF	Associated malignancy	PNP	BO	
1^2^	34/M Bengali	18 months (lichen planus)	PRED HCQ MTX CyA MMF	Oral mucosal ulceration.	IIF rat bladder (+) ICSA (−)	**Oral mucosal:** Lichenoid stomatitis	**Oral mucosal:** Staining with IgG & C3 of basal epidermal ICS and BMZ. Linear band of fibrinogen along BMZ.	MCD: 6 cycles RTX, CYC, HU, vincristine, PRED	MMF TAC Monthly IVIG	Nil	8 years after the diagnosis of PNP – residual oral lichenoid lesions, maintained sequentially on MMF, TAC, and sirolimus for 2 years. Sirolimus subsequently ceased. Serial FDG‐PET & serology unremarkable.
2^2^	63/M Caucasian	1 month (nil)	Nil	Oral mucosal ulceration.	IIF rat bladder (+) ICSA (−) SBMA (−)	**Oral mucosal:** Lichenoid stomatitis	**Oral mucosal:** Staining with IgG & C3 of basal epidermal ICS. Shaggy band of fibrinogen along BMZ.	BCL: 6 cycles RTX, CYC, HU, vincristine, PRED 3‐monthly maintenance RTX for 12 months	Pulsed IV methylprednisolone PRED (up to 1 mg/kg)	Nil	7 years after the diagnosis of PNP – remission, off treatment. Serial FDG‐PET & pulmonary function unremarkable.
3	13/F Caucasian	18 months (lichen planus)	PRED (up to 1 mg/kg) HCQ (200 mg daily)	Oral mucosal ulceration.	IIF rat bladder (+) ICSA (−) SBMA (−) DSG1/3 (−)	**Oral mucosal:** Lichenoid stomatitis	**Oral mucosal:** Staining with IgG of basal epidermal ICS. Shaggy band of fibrinogen along BMZ.	UCD: Resection	PRED (up to 1 mg/kg) HCQ (400 mg daily)	Nil	3 years after the diagnosis of PNP – remission, off treatment. Serial FDG‐PET, HRCT & pulmonary function unremarkable.
4	28/M Fijian Indian	8 months (pemphigus vulgaris)	PRED (up to 1 mg/kg) AZA (1.25 mg/kg) MTX (10 mg weekly)	Oral, perineal mucosal ulceration. Cutaneous hyperpigmented/hyperkeratotic/erythematous papular rash	IIF rat bladder (+) ICSA (+) SBMA (−) DSG1/3 (−)	**Oral mucosal:** Acantholysis **Skin:** Lichenoid inflammation	**Oral mucosal:** Interrupted linear staining with C3 & fibrinogen along BMZ at sites of acantholysis. **Skin:** Staining with IgG of basal epidermal ICS.	UCD: Resection	Pulsed IV methylprednisolone IVIG (2 g/kg – 27 g ×5 doses) PRED (up to 0.6 mg/kg) RTX (1 g ×2 doses)	Nil	7 years after the diagnosis of PNP – remission, off treatment. Serial FDG‐PET, serology & pulmonary function unremarkable.
5	55/F Caucasian	4 months (lichen planus)	PRED (up to 1 mg/kg) HCQ (200 mg daily)	Oral, perineal mucosal ulceration.	IIF rat bladder (+) WB for envoplakin & periplakin antibodies (+) ICSA (−) DSG1/3 (−)	**Oral mucosal:** Lichenoid stomatitis	**Oral mucosal:** Staining with IgG & C3 of basal epidermal ICS and BMZ. Linear band of fibrinogen along BMZ.	BCL: 5 cycles bendamustine, RTX	PRED (up to 1 mg/kg) MMF (6 months, ceased secondary to leukopenia)	Pulsed IV methylprednisolone, 5 cycles plasmapheresis	3 years after the diagnosis of PNP – residual inactive oral lichenoid lesions, on 1 mg of PRED, and maintenance IVIG for secondary hypogammaglobulinemia. Serial HRCT improved.

Abbreviations: AZA, azathioprine; BCL, B‐cell lymphoma; BMZ, basement membrane zone; BO, bronchiolitis obliterans; CyA, cyclosporine; CYC, cyclophosphamide; DSG, desmoglein antibodies; FDG‐PET, fluorodeoxyglucose positron emission tomography; HCQ, hydroxychloroquine; HRCT, high resolution computed tomography; HU, hydroxyurea; ICS, intercellular cement substance; ICSA, intercellular cement substance antibodies; IIF, indirect immunofluorescence; IVIG, intravenous immunoglobulin; MCD, multicentric Castleman disease; MMF, mycophenolate; MTX, methotrexate; PNP, paraneoplastic pemphigus; PRED, prednisolone; RTX, rituximab; SBMA, skin basement membrane antibodies; TAC, tacrolimus; UCD, unicentric Castleman disease; WB, western blot.

## 2 CASES

Amongst the five cases of PNP (Table 1), three were male and two female, with an average age at presentation of 38.6 years (range 13–64 years). All cases were associated with an underlying haematological malignancy, none of which were clinically apparent at the time of the mucocutaneous manifestations and were only detected upon screening for a neoplastic process – multicentric Castleman disease (MCD, *n* = 1), unicentric Castleman disease (UCD, *n* = 2) and B‐cell lymphoma (BCL, *n* = 2). Four cases received an alternate diagnosis prior to their PNP diagnosis in our institute, with all cases having a mean symptom duration of 9.8 months prior to PNP confirmation. In all cases, the diagnosis of PNP was based on clinical, serological and tissue‐based investigation results, including persistent, treatment‐refractory oral/extra‐oral mucocutaneous ulceration, positive serological findings by indirect immunofluorescence (IIF) using rat bladder substrate, and characteristic findings by direct immunofluorescence (DIF) of tissue specimens. Of note, histopathology for each case was non‐specific; this partly may have contributed to delayed PNP diagnosis, particularly for those with the initial diagnosis of LP. Following appropriate therapy of the associated malignancy and PNP, Patients 1–4 (Table 1) achieved clinical and serological remission, and were able to cease immunomodulatory therapy without recurrence of PNP. Patient 5 was also seropositive by Western blot using human epidermal extracts against 210 kDa envoplakin and 190 kDa periplakin (in‐house immunoblot, Kurume University School of Medicine, Japan). Peripheral blood flow cytometry demonstrated a small monoclonal B‐cell population. This prompted discovery of an ill‐defined FDG‐avid pre‐sacral mass on imaging, subsequently diagnosed as a low‐grade BCL by tissue histopathology and flow cytometry. She insidiously developed exertional dyspnoea, with an obstructive ventilatory defect on spirometry and features of BO on HRCT. In addition to treatment of her malignancy and PNP, she received pulsed intravenous methylprednisolone and five plasma exchanges for her BO. Three years on, her PNP and lymphoma remain in remission, and she remains on 1 mg of prednisolone and IVIG for secondary hypogammaglobulinemia.

## 3 DISCUSSION

### 3.1 Red flags for PNP

**FIGURE 1:**
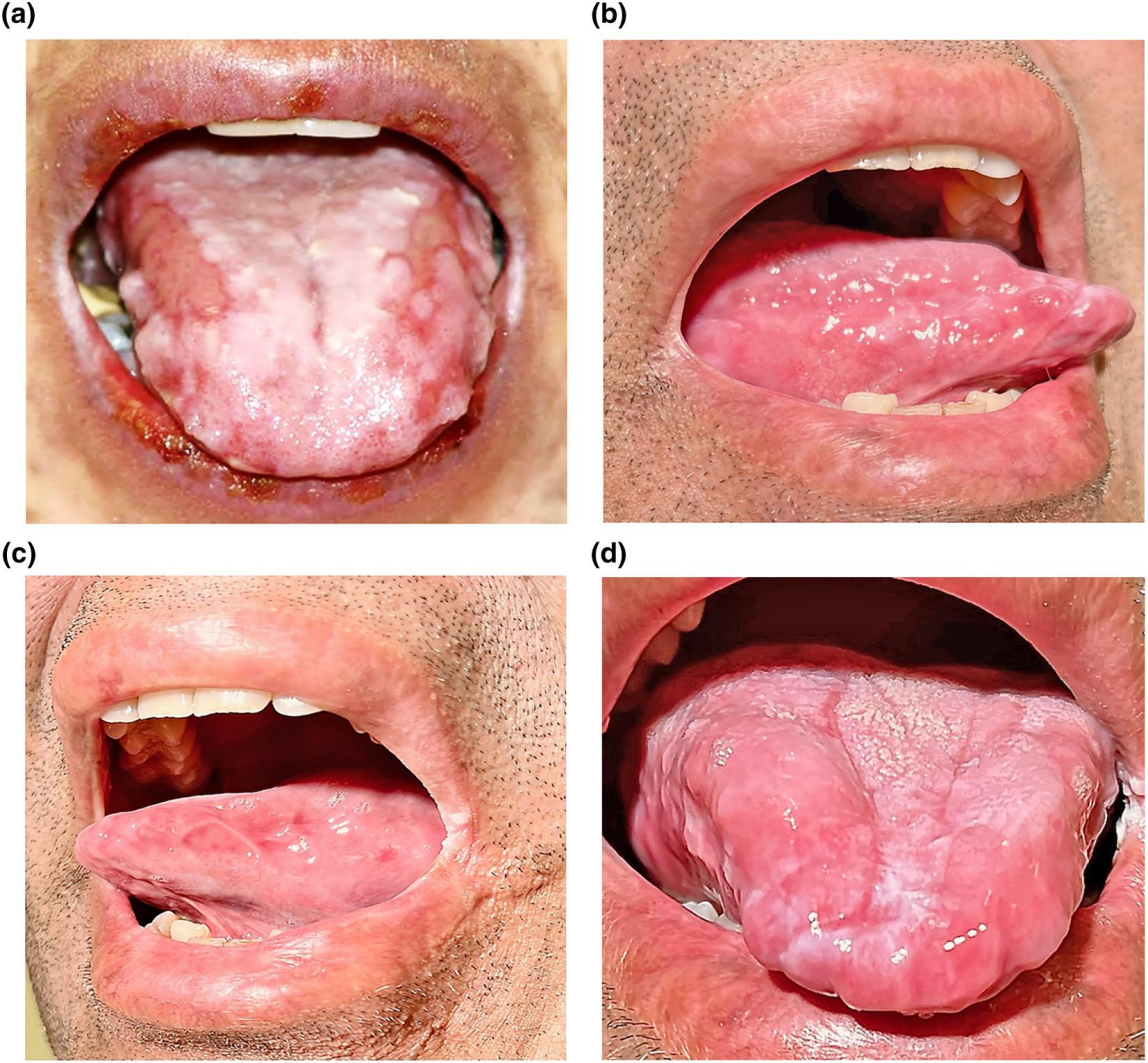
Clinical photos of Patient 1. At the time of diagnosis (a) there was a distinctive pattern of bilateral ulceration and erosion, involvement of the upper and lower lips, typically seen with erythema multiforme, and bilateral ulceration of the latero‐dorsal tongue, as can be seen with oral lichen planus (LP). Nine years later, after surgical resection of the Castleman's disease, resolution of disease was achieved (b‐d), including the right lateral tongue with lips (b), left lateral tongue with lips (c), and a frontal view of the tongue showing features of mild lichen planus with striae and plaques but no mucosal ulceration (d).

Our cases presented similarly with a distinctive patten of treatment‐refractory oral mucosal inflammation and ulceration localised to the latero‐dorsal, tongue and lips (Figure 1), with or without extra‐oral mucocutaneous involvement. The majority had received an alternate diagnosis prior to our tertiary care. Although clinical features may overlap with autoimmune bullous diseases and inflammatory dermatoses, therapy resistance (characteristic in all five cases) and polymorphic cutaneous eruptions (seen in Patient 4) are features that may portend PNP, heightening the index of suspicion in our cases.^3^

**FIGURE 2:**
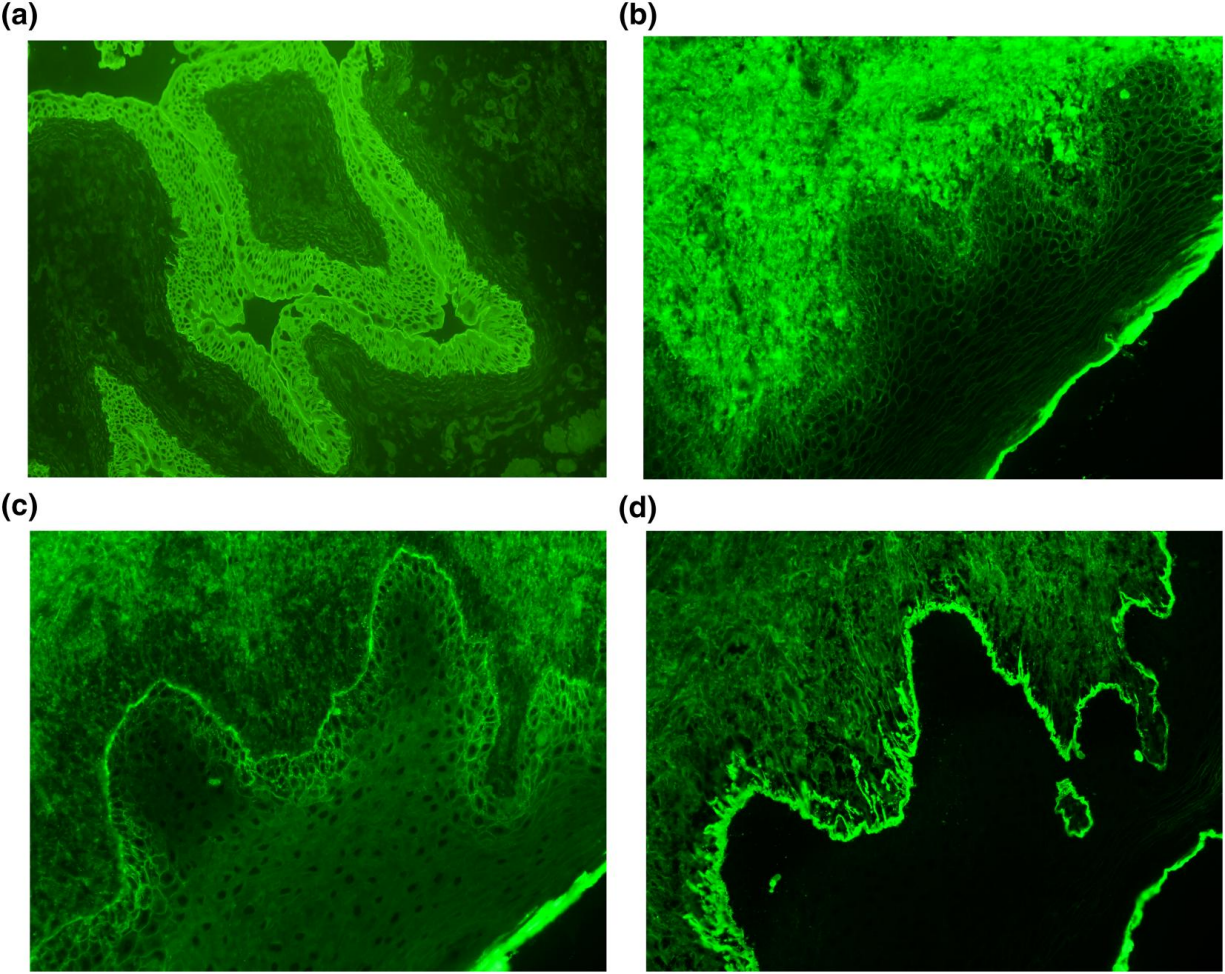
(a) Indirect immunofluorescence microscopy demonstrates IgG autoantibody binding to transitional epithelium on rat bladder substrate (magnification, 200x). (b–d) Direct immunofluorescence of the oral mucosal biopsy of Patient 5 (magnification, 200x). There is IgG deposition along the basal epidermal ICS (b), C3 deposition along the basal epidermal ICS and BMZ (c), and a shaggy fibrinogen band along the BMZ (d).

The pathogenic autoantibodies that underpin PNP are typically directed against plakin family proteins, as well as α2‐macroglobulin‐like antigen‐I, BP180, p200 protein, desmogleins 1 and 3, and desmocollins.^4^ IIF assays utilizing rat bladder substrate, which is rich in plakin proteins, demonstrates staining of the transitional epithelium (Figure 2a) and has a sensitivity of 86% and specificity of 98% for anti‐plakin antibody‐associated PNP; these were positive in all five of our cases.^5^ Immunoblot assays utilizing epidermal extracts of envoplakin and periplakin have demonstrated similarly high sensitivities and specificities, however performance characteristics of commercial blots may be laboratory‐dependent.^5^ ELISAs for anti‐envoplakin and anti‐periplakin antibodies have demonstrated lower sensitivity and specificity than IIF and immunoblot assays.^6^ These factors should be considered during investigation.

Histopathology varies with lesion morphology – blisters or erosions may show suprabasal acantholysis and detachment, and inflammatory maculopapular lesions may show dyskeratotic or necrotic keratinocytes, vacuolar interface dermatitis or lichenoid infiltrates.^5^ DIF findings are key to the diagnosis. There is often overlap with PV and LP, with the combination of IgG and C3 complement deposition limited to the basal epidermal intercellular surface (ICS), and fibrinogen deposition along the basement membrane zone (BMZ) (Figure 2b–d).

### 3.2 Malignancy screening & treatment

Malignancies may be occult and tend to be haematological, mostly B‐cell neoplasms or Castleman's disease, as was in our five cases.^7^ Therefore, findings consistent with PNP should prompt screening with a CT and flow cytometry of peripheral blood as a starting point. A minority of patients may not have an underlying malignancy, however should be closely monitored for its subsequent development.^7^

Treatment of benign tumours or localised Castleman's disease can lead to disease remission following tumour resection.^8^ Whilst all our patients achieved PNP remission following treatment of their malignancy, this may not always result. A combination of treatments that target humoral and cellular immunity may be appropriate, although this may depend on the underlying malignancy.

### 3.3 Bronchiolitis obliterans

PNP is associated with a poor prognosis, which is not only a reflection of malignancy or immunosuppression, but also BO, which may occur in up to 30% of PNP patients,^1^ has a mortality rate of at least 40%,^7^ and therefore must be screened for in all patients. It may present insidiously with dyspnoea or cough, an obstructive defect on pulmonary function testing and air trapping on CT,^1^ and its irreversible course may progress independent of the malignancy or PNP. Although its pathogenesis is not well defined, it is thought that the autoantibodies target certain plakin proteins expressed in respiratory epithelia,^9^ which was likely the case in Patient 5, who was seropositive for anti‐envoplakin and anti‐periplakin antibodies. She was managed initially with plasma exchange – for the putative role of the autoantibodies in the disease pathophysiology – systemic corticosteroids and maintenance immunosuppression. Although BO confers a poor prognosis and is resistant to immunosuppressive medications,^8^, ^10^ our case experienced stabilization over a prolonged duration following treatment, which may have been the outcome of diagnostic vigilance and early aggressive management.

## 4 CONCLUSION

**FIGURE 3:**
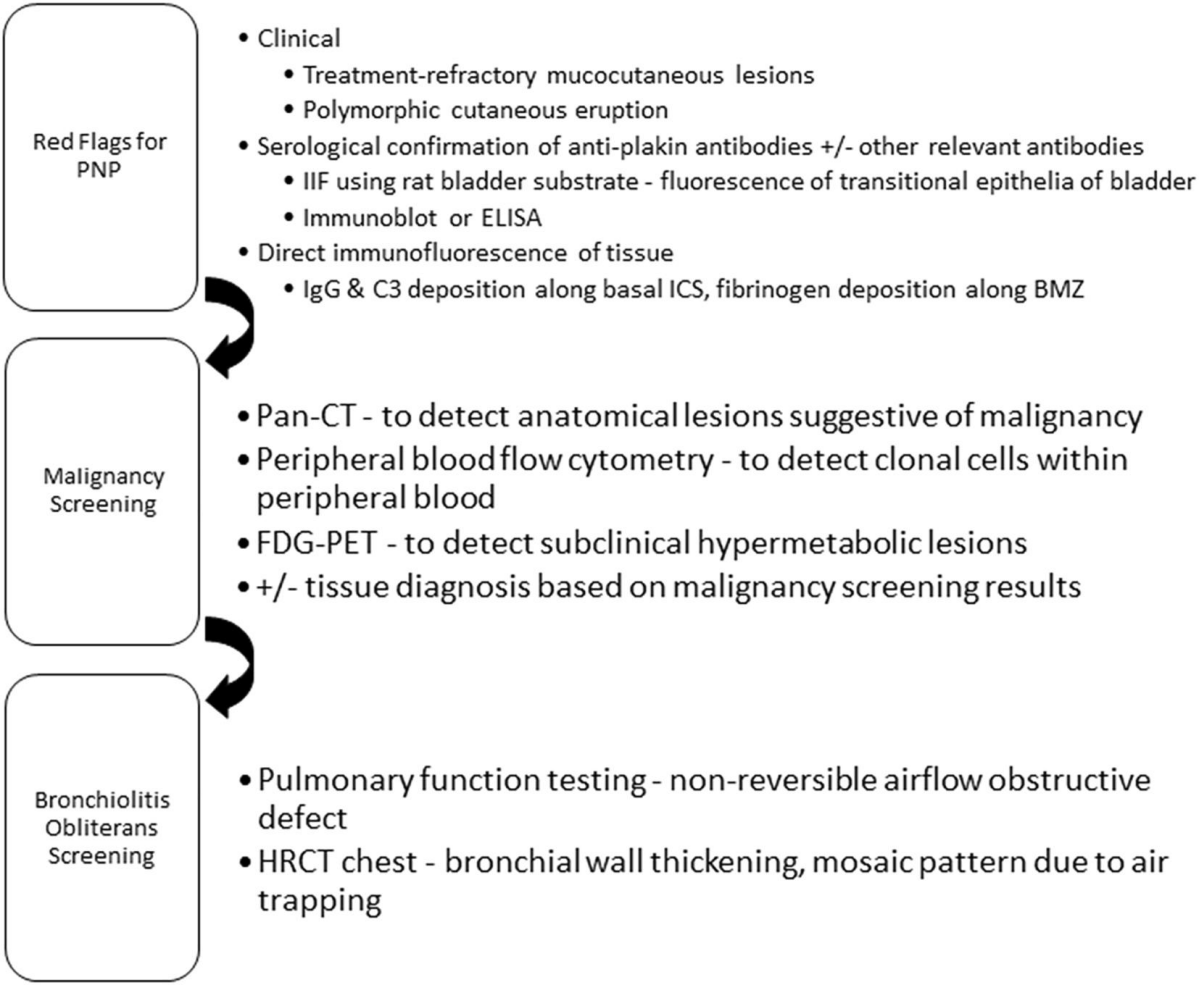
Approach to paraneoplastic pemphigus

Good clinicopathologic correlation is key to the timely diagnosis of PNP (Figure 3), supported by the detection of PNP antibodies on testing that includes IIF on rat bladder substrate in preference to other serological assays.^11^ Tissue histopathology and DIF definitively establishes the diagnosis of PNP, and should prompt further investigation for an underlying neoplasm, even in the absence of suggestive symptoms. BO is a life‐threatening complication of PNP, and follows a course independent of the malignancy and mucocutaneous disease; judicious disease monitoring for its occurrence should be considered even in the absence of symptoms. Treatment of the malignancy may lead to remission of the PNP without the need for persistent immunosuppression.

As demonstrated in this small series, early clinical suspicion, extensive imaging to investigate for often clinically silent malignancy, intensive multidisciplinary care, awareness for and management of BO, if present, resulted in impressive long‐term survival when compared with recent data of less than 50% survival at 6 years^12^

## AUTHOR CONTRIBUTIONS

**Jonathan S. Emerson**: Conceptualization (Lead); Data curation (Lead); Methodology (Equal); Writing – original draft (Lead); Writing – review & editing (Lead). **Mark Schifter**: Data curation (Equal); Methodology (Equal); Writing – original draft (Supporting); Writing – review & editing (Supporting). **Pei Dai**: Data curation (Equal); Methodology (Equal); Writing – original draft (Supporting); Writing – review & editing (Supporting). **Jocelyn Jiang**: Data curation (Equal); Methodology (Equal); Writing – original draft (Supporting); Writing – review & editing (Supporting). **Mark S. Taylor**: Data curation (Equal); Methodology (Equal); Writing – original draft (Supporting); Writing – review & editing (Supporting). **Michelle Kang**: Data curation (Equal); Methodology (Equal); Writing – original draft (Supporting); Writing – review & editing (Supporting). **Kenelm Kwong**: Data curation (Equal); Methodology (Equal); Writing – original draft (Supporting); Writing – review & editing (Supporting).  **Hadleigh Clark**: Data curation (Equal); Methodology (Equal); Writing – original draft (Supporting); Writing – review & editing (Supporting). **Suzanne Culican**: Data curation (Supporting); Methodology (Equal); Writing – original draft (Supporting); Writing – review & editing (Supporting).  **David Campbell**: Data curation (Supporting); Methodology (Equal); Writing – original draft (Supporting); Writing – review & editing (Supporting). **Ming‐Wei Lin**: Conceptualization (Lead); Data curation (Lead); Methodology (Lead); Supervision (Equal); Writing – original draft (Equal); Writing – review & editing (Equal).

## CONFLICT OF INTEREST

The authors declare that they have no conflict of interest.

## ETHICS STATEMENT

Patients have consented to the publication of the cases, including publishing of photographs and data.

## Data Availability

Data sharing is not applicable to this article as no new data were created or analyzed in this study.
